# Physiological strategies explain mortality differences among ecologically and culturally significant Australian desert plants following a hotter drought

**DOI:** 10.1093/conphys/coag079

**Published:** 2026-10-28

**Authors:** Jaylen D Nicholson, Rita Okai, Muitjulu Mala Rangers, Andy Leigh, Danielle A Way, Nicholas A Macgregor, Rebekah Robertson, Samuel D Merson, Tracey Guest, Adrienne B Nicotra

**Affiliations:** Research School of Biology, Australian National University, 46 Sullivans Creek Road, Canberra, Australian Capital Territory 0200, Australia; Uluu-Kata Tjua National Park, Parks Australia, 1680 Uluu Road, Yulara, Northern Territory 0872, Australia; Muitjulu Community Aboriginal Corporation, Muitjulu Community, Muitjulu, Northern Territory 0872, Australia; School of Life Sciences, University of Technology Sydney, 15 Broadway, Ultimo, New South Wales 2007, Australia; Research School of Biology, Australian National University, 46 Sullivans Creek Road, Canberra, Australian Capital Territory 0200, Australia; Department of Biology, University of Western Ontario, 1960 Middlesex Drive, London, Ontario N6A 3K7, Canada; Nicholas School of the Environment, Duke University, 9 Circuit Drive, Durham, NC 27708, USA; Parks Australia, Department of Climate Change, Energy, the Environment and Water, Canberra, Australian Capital Territory 2601 Australia; Durrell Institute of Conservation and Ecology, University of Kent, 5 Woodland Way, Canterbury, Kent CT2 7NZ, UK; Uluu-Kata Tjua National Park, Parks Australia, 1680 Uluu Road, Yulara, Northern Territory 0872, Australia; Parks Australia, Department of Climate Change, Energy, the Environment and Water, Canberra, Australian Capital Territory 2601 Australia; Uluu-Kata Tjua National Park, Parks Australia, 1680 Uluu Road, Yulara, Northern Territory 0872, Australia; Research School of Biology, Australian National University, 46 Sullivans Creek Road, Canberra, Australian Capital Territory 0200, Australia

**Keywords:** Arid, Australia, climate change, culturally important, dieback, drought, heatwave, indigenous knowledge, leaf traits, plant physiology

## Abstract

Climate change-induced drought and heatwave events (hotter droughts) are causing mass plant dieback events globally. Recently, Uluru-Kata Tjuta National Park (UKTNP) in central Australia experienced a widespread plant dieback (mortality) event. Anangu (Traditional Owners) are concerned about the resulting negative impacts to ecosystems and wish to understand more about the underlying causes of mass plant death. With a focus on ecologically and culturally significant species chosen by Anangu, we measured morphological and physiological traits to characterize differences in physiological strategy underpinning the patterns of dieback observed at UKTNP. Maintenance of leaf relative water content (RWC) was the leaf trait that best predicted dieback severity, with all species that had low levels of dieback exhibiting drought-avoidance strategies where RWC was maintained between spring and summer. Most species with moderate and high levels of dieback exhibited drought-tolerance strategies, evidenced by large declines in seasonal RWC and higher wood densities. However, two small shrub species with high dieback severity may have died due to failure of different physiological mechanisms—one of hydraulic failure via drought tolerance and one of carbon limitation via drought avoidance—highlighting the importance of considering species-specific trait combinations to understand drivers of mortality. Understanding the drivers of dieback will help to support Anangu-led land stewardship and conserve the natural and cultural values of this important landscape.

Pitjantjatjara abstract: Kuwari, punu munu ukiri tjuta piltiringanyi manta winkingka panya ailurungka munu kuli pulkangka. Mungatu, Uluru-Kata Tjuta National Parkangka, ailuru pulkangku piltira wiyanu punu munu ukiri tjuta Anangu tjutaku. Nganana mukuringanyi nintiringkutjaku nyaa nguru punu munu ukiri tjuta wiyaringu, ka punu kutjupa tjuta palyatu ngaranyi. Nganana kulini punu munu ukiri wankatu ngaranyi panya kapi ngarinyi nyarlpingka. Tjingurula kulini punu pilti tjutangku putu kapi Kanyini nyarlpingka. Tjinguru kapi putu pakani nyarlpinguru. Pukara munu kaliny-kalinypa tjuta wiyaringu ailurungka. Tjinguru pukarangku kapi kawalanu aturu ungu. Tjinguru kaliny-kalinypangku mai munu tjuratja kawalanu aturu ungu. Nyangatjalanya nintini panya punu wiyaringanyi nyanga paluru tjanalanguru, punu kutjupa tjuta tjinguru wiyaringanyi kutjupa kutjupanguru. Nganana tjungu warkarira nintiringkutjaku panya ailurungku ngura nganampa piltini. Nyanga palulanguru nganana ngura nganampa wirura atunmangkuntjaku.

## Introduction

The frequency of mass dieback (including mortality) in plant communities is increasing globally (Luna-Aranguré *et al.,* 2025). Dieback is a complex physiological process and can involve several contributing or interacting factors, making it difficult to determine the causes of decline in individual cases (Allen, 2009). However, drought and heatwaves are known to be one of the most widespread drivers of mass plant death. In water-limited environments, local extreme heat can combine with severe drought to cause rapid plant mortality (Choat *et al.,* 2018). Anthropogenic climate change is causing a greater frequency of more intense drought and heatwave events (i.e. hotter droughts), increasing the likelihood of more frequent and widespread dieback (Bauman *et al.,* 2022; Hartmann *et al.,* 2022; McDowell *et al.,* 2022).

Global data show that as aridity and temperature increase across broad environmental gradients, plant traits (both across and within species) change, often in a concerted manner (Niinemets, 2001). Generally, leaf area decreases whilst leaf mass per area (LMA) and leaf dry matter content (LDMC) increase, reflecting global convergence on a more conservative leaf structure to minimize water loss and maintain leaf function under dry and hot conditions (Niinemets, 2001; Li and Prentice, 2024). Furthermore, higher wood density (WD) and narrower xylem vessels act to maintain water transport and reduce the risk of embolism under drier soil conditions (Hacke *et al.,* 2001). However, a variety of strategies with different trait combinations allow plants to exist across a range of environments (Ackerly, 2004; Laughlin *et al.,* 2023).

The strategies that are particularly relevant for arid ecosystems can be conceptualized as ‘drought escape’, drought avoidance’ and ‘drought tolerance’. A drought escape strategy dictates that plants complete their life cycle within the window of favourable conditions, as exemplified by desert ephemeral plants with exceptionally high CO_2_ assimilation rates (Mooney *et al.,* 1976). Drought-avoiding plants have characteristics such as deep root systems to access more abundant water sources, stem succulence which confers greater internal water storage capacity (e.g. *Disphyma crassifolium*, see Guo *et al.,* 2023), and sunken stomata (e.g. *Grevillea striata*, see Jordan *et al.,* 2008) and curled leaves (e.g. spinifex species such as *Triodia schinzii*, see Winkworth, 1967), which decrease stomatal water losses (Fang and Xiong, 2015). Often, under prolonged dry conditions, drought-avoiding species exhibit conservative water use (*sensu* isohydry or stronger stomatal sensitivity to drying soil), which limits water losses and means that these plants typically operate within a narrower range of leaf water potentials and do not reach the extreme negative water potentials of drought tolerating species (Choat *et al.,* 2012; Martínez-Vilalta and Garcia-Forner, 2017). The avoidance strategy thus prioritizes hydraulic safety over carbon assimilation, leading to reduced assimilation during extended drought (McDowell *et al.,* 2022). Drought-tolerating plants, in contrast, maintain assimilation during prolonged dry conditions via adaptations such as high WD and small, thick leaves that reduce water loss while maintaining stomatal conductance which also prevents extreme leaf temperatures (Choat *et al.,* 2012). The relatively less conservative stomatal behaviour under low soil moisture (*sensu* anisohydry e.g. *Acacia aptaneura*, see Nolan *et al.,* 2017) can lead to more variable leaf relative water content (RWC) than species that cease water use under low soil moisture. During mild stress, drought-tolerating species are able to maintain function at a much broader range of conditions than drought-avoiding species (Choat *et al.,* 2018).

Although these drought avoidance and drought tolerance frameworks provide useful mechanistic descriptions of coordination in physiological and morphological traits, these strategies do not imply universally lower or higher drought-induced mortality risk, making predicting mortality from traits difficult (Trugman *et al.,* 2021). Hydraulic failure is a well-established pathway of drought-induced mortality, whereby excessive xylem tension causes embolism and loss of hydraulic function (Choat *et al.,* 2018; McDowell *et al.,* 2022). However, species with greater resistance to embolism do not consistently experience lower mortality (Jacobsen and Pratt, 2018). For example, temperate deciduous tree and shrub species with the greatest canopy dieback and native embolism tended to be species with highest wood densities (Hoffmann *et al.,* 2011), which would be assumed to have the highest resistance to embolism (Hacke *et al.,* 2001). Similarly, in a Californian chaparral shrubland, drought-induced mortality was higher among species with more cavitation-resistant xylem, despite greater resistance to hydraulic failure (Paddock *et al.,* 2013). Carbon limitation has also been proposed as a pathway of drought-induced mortality (McDowell *et al.,* 2008, 2022), although its importance as an independent cause of mortality remains contested (Hartmann, 2015; Adams *et al.,* 2017). Prolonged drought limiting carbon assimilation prior to mortality has been observed in few species, with two prominent examples in piñon pine (*Pinus edulis* Engelm., Breshears *et al.,* 2009) and in ponderosa pine (*Pinus ponderosa* Douglas ex C. Lawson, McDowell *et al.,* 2010). Overall, more studies are needed to establish the trait correlates of drought avoidance and tolerance and how these strategies influence the physiological pathways leading to mortality.

The combined effects of heat and drought stress can alter the relationship between physiological strategy and mortality risk, as stomatal responses that conserve water under drought (i.e. drought avoidance) can shift towards increased water loss under combined extreme heat and drought to prevent leaf overheating (Marchin *et al.,* 2022). Thus, it remains unclear whether broad drought-response strategies confer greater or lower mortality risk when drought is accompanied by extreme heat. Ecosystems where variable and species-specific mortality has occurred provide an opportunity to examine coordinated variation in plant water-use behaviour traits, such as RWC and stomatal regulation, as well as leaf and wood structural traits, to better characterize broad physiological strategies and improve our understanding of the mechanisms underlying dieback under increasingly frequent and severe hotter droughts (Pivovaroff *et al.,* 2016).

After a record-breaking drought and heatwave event in 2018–2019, Uluru-Kata Tjuta National Park (UKTNP) in the deserts of Australia’s Northern Territory experienced a mass mortality, dieback event (Wright *et al.,* 2023). Annual rainfall for 2019 was the lowest recorded at only 27 mm (38-year average rainfall is 269 mm Bureau of Meteorology, 2025) coinciding with several weeks in summer with temperatures above 45°C. Anangu (Pitjantjatjara and Yankunytjatjara people, the Traditional Owners) expressed their concern and a desire to understand why plants were dying on Country (Country is a term used by Aboriginal peoples to describe the lands, seas, and waterways, including cultural and spiritual connection, belief, law, language, and identity.) A mortality study on a range of culturally and ecologically significant species at UKTNP revealed that: (i) some species had very high mortality (>90%), whereas some had very low mortality (0%); (ii) there was generally greater survival in larger individuals; and (iii) there were density-dependent mortality relationships that differed between species (Wright *et al.,* 2023). A follow-up study investigating landscape-level patterns in dieback suggested that species-specific mortality patterns across UKTNP were associated with topographic trends across dune sequences, and that some are linked to stand density and size of individuals (Godfree and Knerr, 2025). However, the drivers and physiological mechanisms underpinning dieback in these culturally important species within UKTNP remain unknown.

The ecocultural impacts of climate-induced mortality, particularly for First Nations peoples, are only recently garnering attention. Loss of stewardship following colonization, in addition to altered ecosystem processes from changed fire practices, invasive species and climate change, lead to disproportionate impacts on Indigenous Peoples and their Country (Wickham *et al.,* 2022; Hankins, 2024). UKTNP is a site of immense importance for Anangu. Thus, rather than study the rare or threatened species that western conservation management may consider, our team, which includes Anangu Traditional Owners and national park rangers, selected a range of species that are ecologically and culturally significant (e.g. including dominant species involved in key ecological processes and functions such as habitat provision for fauna, and/or those holding other high cultural value). Understanding why and where these species are dying will allow Anangu to have a more holistic approach to managing Country and is of critical importance to conserving natural and cultural values and understanding how to maintain functional ecosystems in times of rapid ecological change.

Working on naturally occurring individuals in a range of vegetation types, we assessed a suite of morphological (LMA, LDMC, leaf thickness, leaf area and WD) and physiological parameters (RWC, carbon stable isotope fractionation, carbon:nitrogen ratio) (simply referred to as traits hereafter) hypothesized to confer the ability to tolerate or avoid heat and water stress. Our objectives were to: (i) determine the traits or combinations of traits associated with observed dieback; (ii) understand whether those traits vary spatially among broad habitat types or temporally between seasons within an individual; and then (iii) elucidate the drought tolerance or avoidance strategy that each species employs to help explain the observed patterns of mortality. Overall, we predicted that species with drought-tolerating strategies would show greater leaf trait variability across habitats and seasons, especially in RWC, while drought-avoiding species would maintain more stable trait values across space and time.

## Materials and Methods

### Study area

Uluru-Kata Tjuta National Park is located in the central desert region of Australia’s Northern Territory (Fig. 1), 300 km southwest of Alice Springs. The climate is classified as Grassland hot (persistently dry) under the Köppen climate types (Peel *et al.,* 2007). It has a mean maximum temperature of 30.3°C and a mean minimum temperature of 14.2°C (Bureau of Meteorology, 2025). Mean annual rainfall for Yulara airport (20 km from UKTNP) is 269 mm and median rainfall is 222.5 mm, indicating that interannual rainfall variability is high. Summers (Novembe–March) are hot and usually wetter, with maximum temperatures exceeding 40°C for days and even weeks at a time. Winters are cool and dry with daytime temperatures at 20°C, with a slow gradual increase in daily temperatures until summer. A deep, red siliceous sand dune field vegetation complex dominates the majority of UKTNP, intersected by creek lines and gravel outwash plains. Unlike many dune systems worldwide, the dune field complex at UKTNP is vegetated and therefore not subject to short-term and large-scale dune movement from aeolian processes (Keith, 2017; Morton, 2022).

**Figure 1:**
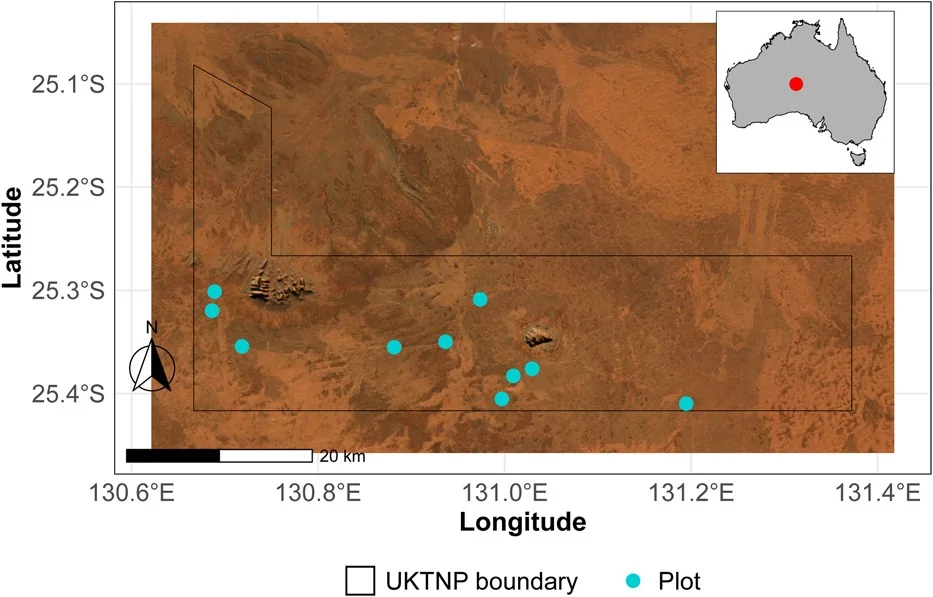
Location of Uluru-Kata Tjuta National Park in Australia’s Northern Territory (inset) and the location of the ten study plots.

Anangu have identified three main habitat types in the dune field complex of UKTNP: tali (sand dune), pila (sand plain) and puti (woodland/scrubland) (Table 1). Soil moisture, nutrients and particle size vary across the habitat types, associated with a catenary sequence from top of the tali, into the pila, with puti being the most low-lying point in the dune fields. Briefly, soil moisture and fractions of silt and clay increase from the top of tali, to pila, to puti (Table 1, Buckley, 1982. See Supplementary Material for further details of edaphic properties).

**Table 1:** Description of habitat types sampled

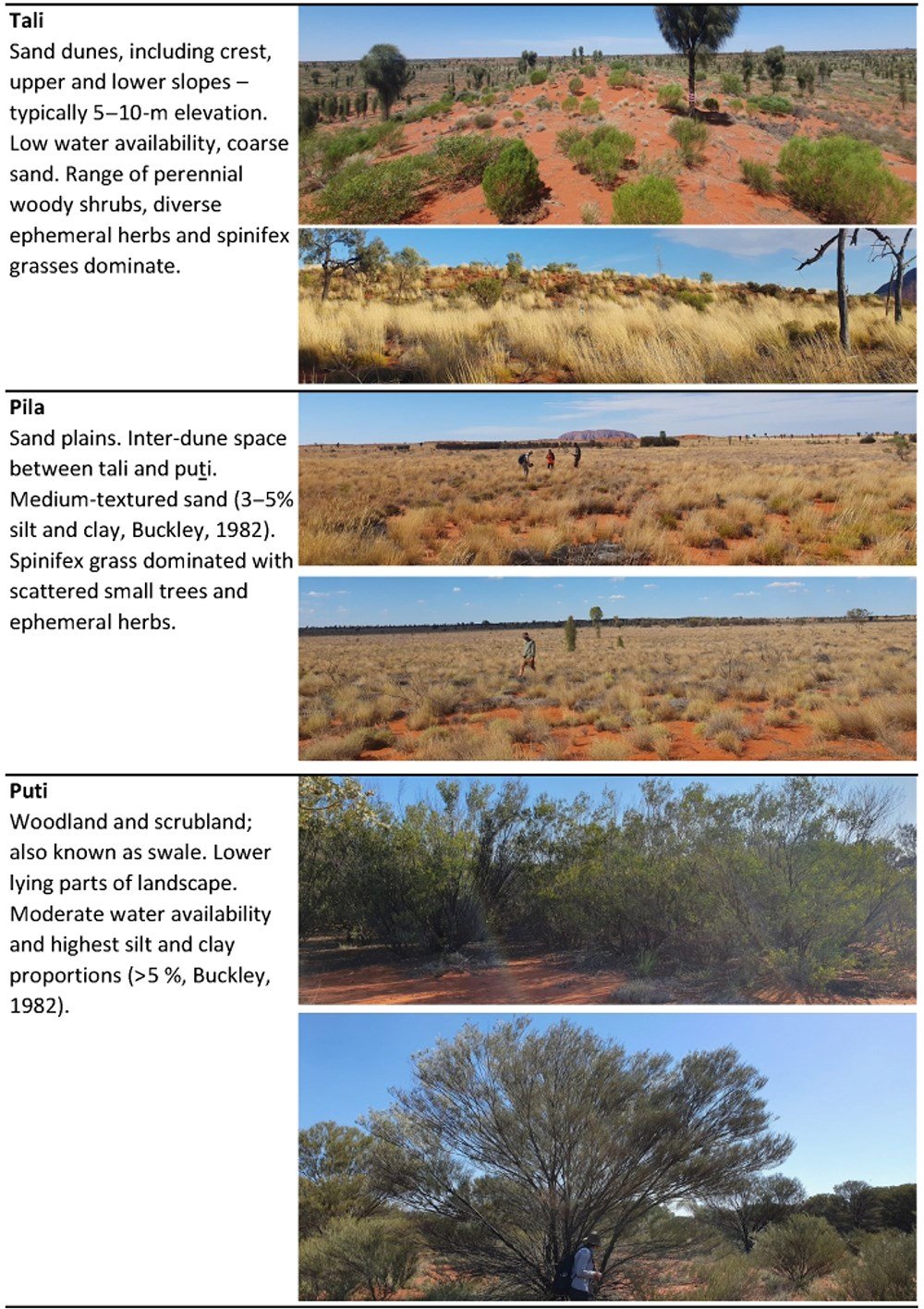

### Study species

We named this study Punu Tjuta—meaning many plants in Pitjantjatjara. Nine species were chosen with Anangu over many consultations and field trips on Country. The plants chosen reflected those that Anangu are concerned about, want to know more about, and that hold cultural and ecological importance (Table 2; Pitjantjatjara names used hereafter). The study species also represent the range of lifeforms and dominant plants present in the dune field vegetation complex, ranging from spinifex grass of the pila (sand plain) and tali (sand dune) through to large trees on the puti (*Acacia* woodland).

**Table 2:** List of plant species surveyed at Uluru-Kata Tjuta National Park, ranked by mortality rate as quantified by Wright *et al.* (2023)

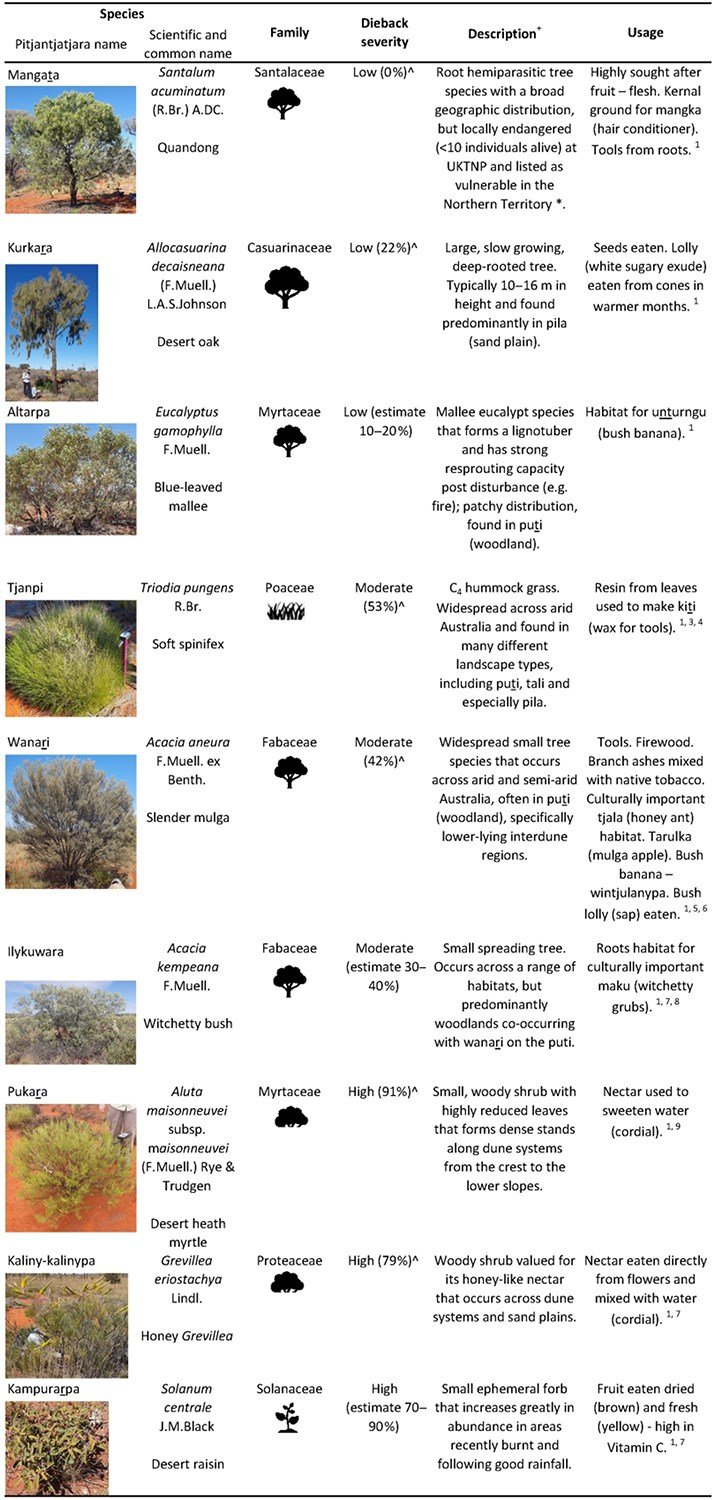

For descriptions of habitats, see Table 1. Mortality class was determined from Wright *et al.* (2023) data and on-ground observations.

^ Percentage mortality from Wright *et al.* (2023).

+ Description adapted from Flora NT (https://eflora.nt.gov.au/).

^*^ Department of Environment, Parks and Water Resources (2021).

^1^R. Okai (pers. comms.), ^2^O’Connell *et al.* (1983), ^3^Gamage *et al.* (2012), ^4^Cane (1987), ^5^Cleland and Tindale (1959), ^6^Walsh (1990), ^7^Latz (1995), ^8^Kalotas (1983), ^9^Ward *et al.* (2023).

The selected species include some that exhibited high mortality rates across UKTNP after the hotter drought in 2019 and some that did not. Mortality rates for six of the nine species were assessed 1.5 years after the peak of the hotter drought in 2021 (Wright *et al.,* 2023). Mortality rates varied across the species, with low rates for mangata (*Santalum acuminatum*, 0%) and kurkara (*Allocasuarina decaisneana*, 22 ± 7%; means ± SE) and moderate rates for wanari (*Acacia aneura*, 42 ± 11%) and tjanpi (*Triodia pungens*, 53 ± 11%). The species exhibiting the highest mortality rates were kaliny-kalinypa (*Grevillea eriostachya*, 79 ± 9%) and pukara (*Aluta maisonneuvei* subsp. *maisonneuvei*, 91 ± 5%, (Wright *et al.,* 2023, Table 2). Taking expert guidance from Anangu and other researchers at UKTNP (Wright *et al.,* 2023; Godfree and Knerr, 2025), we estimated dieback severity for the three remaining species; kampurarpa (*Solanum centrale*), ilykuwara (*Acacia kempeana*) and altarpa (*Eucalyptus gamophylla*). Based on our observations and preliminary landscape-scale dieback severity data (Godfree and Knerr, 2025, and Godfree *et al.* manuscript in preparation), it seemed unlikely that ilykuwara had either very high or very low mortality, so we classified it with moderate dieback severity. Furthermore, ilykuwara is an acacia and is similar in habit to two of the study species considered by Wright *et al.* (2023): *Acacia melleodora* that had a mortality rate of 65% and *Acacia maitlandii* 67%. We estimated altarpa to have low mortality based on landscape-scale imagery (Godfree and Knerr, 2025) and based on our own observations that there were very few recently dead individuals across the landscape that were not clearly killed by fire. Drought mortality is usually associated with retention of some dead leaves on branchlets and/or presence of fine branchlets and limited deeply cracked bark and exposed aged sapwood. Kampurarpa is a small ephemeral herb that germinates from seed and grows rapidly after fire. We inferred that it would likely have had low drought tolerance and consequently high mortality across UKTNP during the hotter drought.

Some species are present in all three habitat types (e.g. tjanpi, altarpa), whereas others are restricted to a single habitat (e.g. ilykuwara). Given the differences in soil moisture, nutrients and particle size between habitat types (Buckley, 1982), and to further explain spatial variability in mortality identified by Wright *et al.* (2023), we designed a sampling regime to capture differences across the landscape in mortality rate both within and between species (Table 2).

### Study design

Eight plots were selected from thirty 100-hectare (1 × 1 km) survey plots established by CSIRO and Parks Australia in 2022 (Godfree and Knerr, 2025). Plots were chosen to (i) include all three habitat types, (ii) maximize the number of target species, (iii) capture the west–east extent of UKTNP, and (iv) allow access on foot. Recently burnt areas (≤3 years) were excluded using fire scar maps from North Australia & Rangelands Fire Information (NAFI, 2025). Within each plot, sampling was stratified by habitat, with three-five individuals per species-habitat combination (except mangata; see below), aiming for minimum 40 individuals per species (Table S1). Sampling area within each plot varied from 1 to 30 ha to maintain similar replication across species despite uneven distributions between plots. Two additional single-habitat plots were included to increase representation of species more restricted to one habitat (e.g. wanari, ilykuwara), totalling 10 plots overall. Mangata, which has only nine known live individuals in UKTNP, was sampled independently.

Field surveys were conducted in spring (September) 2023 and summer (March) 2024 to capture mild versus extreme seasonal conditions. Volumetric soil moisture (0–5 cm depth) was measured at three locations within the crown perimeter of each individual using a FieldScout TDR 350 (Spectrum Technologies, USA) during both campaigns (except one plot in summer 2024 due to instrument failure).

Weather preceding the spring 2023 sampling was mild (maximums < 30°C) with atypically high winter rainfall (June 2023; Fig. S1a), suggesting low physiological stress despite dry surface soils (Fig. S1b). In contrast, weather preceding the summer 2024 trip featured prolonged heat (>40°C) and low rainfall, followed by small rain events during sampling that raised surface soil moisture from 0.9% to 3.1%. Soil moisture was consistently lowest in tali (sand dune) compared with pila (sand plain) and puti (woodland) habitats (Fig. S1b).

### Plant and trait measurements

To limit trait variation artefacts introduced when sampling, we selected adult plants with no marked damage (e.g. from camel grazing or strong wind), or severe insect or pathogen damage. All individuals were located more than 50 m from roads to avoid hydrological edge effects. During the first campaign, plant height and stem diameter (woody species) or canopy dimensions (non-woody) were recorded. A relative size score was then created by log-transforming a volumetric size proxy (basal area * height for woody species; canopy area * height for non-woody species), then standardizing within each species to a *z*-score, allowing size comparisons across species. Each individual was assigned a six-level relative health score (0.5–3, in 0.5 increments) based on visible symptoms such as leaf yellowing, necrosis, or defoliation. Leaves (including phyllodes and cladodes) were collected between 8 am and 11 am using secateurs or a pole pruner, from one plot per day. To standardize light exposure and leaf age, the newest fully expanded, north-facing adult leaves were selected. For each individual, 10–20 leaves were collected and stored in sealed bags within a cooler. For pukara, branchlets containing several hundred small, scale-like leaves were collected.

Samples were processed within 2–4 h of collection. Three leaves per individual were trimmed with a razor and weighed fresh, with leaf thickness measured using digital callipers (avoiding the midrib) and leaf area with the Easy Leaf Area app (Easlon and Bloom, 2014). Leaves were rehydrated overnight (12–16 h) before reweighing for rehydrated mass; preliminary trials showed six hours was insufficient for full rehydration in some species. For pukara, average fresh mass, area, and rehydrated mass were based on 30–100 leaves. Samples were pre-dried in the field (2 h) to prevent spoilage and oven-dried at 105°C for 48 h at the Australian National University for dry mass determination.

All calculations were conducted on an average per leaf basis, whereby the cumulative weight (fresh, rehydrated or dry) and area were divided by the number of leaves per individual. LMA (Table 3) was calculated by dividing mean leaf area by mean dry mass (g m^−2^). Leaf dry matter content (LDMC, Table 3) was calculated as the ratio of dry mass to rehydrated mass. Leaf RWC (Table 3) was calculated using the following formula:

**Table 3:** Plant traits measured for each individual.

**Trait**	**Description**	**Frequency of sampling**
Morphological traits		
Leaf area; LA (cm^2^)	Total one-sided surface area of a leaf. Greater area reflects greater light interception for photosynthesis but increased potential water losses (Niinemets, 2010)	Both seasons
Leaf mass per area; Leaf mass per area; LMA (g m^−2^)	Dry mass divided by the one-sided area of a leaf. Higher LMA reflects greater structural investment (Poorter *et al.,* 2009).	Both seasons
Leaf thickness (mm)	Distance between upper and lower leaf surfaces. Linked to specific adaptations such as photosynthetic capacity and efficiency, and temperature and water regulation (John *et al.,* 2017).	Both seasons
Leaf dry matter content; Leaf dry matter content; LDMC (ratio)	Ratio of leaf dry mass to leaf rehydrated mass. Higher values reflect greater investment into structural leaf components (John *et al.,* 2017).	Both seasons
Wood density; Wood density; WD (g m^−3^)	Wood mass per volume. Higher WD indicates greater ability to tolerate higher water tensions (Chave *et al.,* 2009).	Spring 2023 only
Physiological traits		
Leaf relative water content; RWC (%)	Proportion of leaf water under field conditions relative to the fully hydrated leaf. Indicator of water stress and water regulation strategy (Bartlett *et al.,* 2012).	Both seasons
Leaf carbon isotope fractionation; δ^13^C (‰)	Ratio of the stable isotopes of C. Proxy for water use efficiency across the whole lifespan of a leaf (Seibt *et al.,* 2008).	Spring 2023 only
Leaf carbon-to-nitrogen ratio; C:N	Ratio of C to N in leaves. Reflects structural investment, growth capacity and nitrogen use efficiency (Reich, 2014).	Spring 2023 only

A description of the trait and its ecophysiological significance as well as the sampling frequency are given.

RWC (%) = ((FM − DM)/(TM − DM)) × 100.

where FM is fresh mass, DM is dry mass and TM is turgid mass (or rehydrated mass).

Between three and ten leaves per individual were placed in envelopes for carbon stable isotope and C:N ratio analysis (Table 3). A subset of individuals from each species were selected (10–15 per species, with 3–5 per habitat type). Samples were dried at 80°C for 48 hours prior to being delivered to the Stable Isotope Laboratory at the Research School of Biology at the Australian National University for mass spectrometry and elemental analysis. Carbon stable isotope and C:N ratio analyses were only conducted for the spring 2023 sampling regime.

To standardize across a range of woody lifeforms (from shrubs to large trees), WD (Table 3) was measured on stem segments 1–1.5 cm in diameter. For smaller shrub species, this represented more basal wood, while for larger trees, samples were from terminal branches. One individual per species per habitat was selected per plot. Using secateurs or pole pruners, a 10-cm-long segment was collected, sealed in a zip-lock bag, and stored in a cooler. Wood density was measured using the water displacement method (Pérez-Harguindeguy *et al.,* 2016). Each segment was trimmed to ~ 2.5 cm in length, with its fresh mass when submerged equalling its volume. Samples were then dried at 105°C for 48 hours and reweighed. Wood density (g cm^−3^) was calculated as dry mass divided by volume. Wood density was measured only during spring 2023 sampling. A summary of all trait values measured for all species is found in Table 4.

**Table 4:** Trait means (± standard error) for each species (Pitjantjatjara name and scientific name)

**Species**	**RWC**	**LDMC**	**Area (cm** ^**2**^ **)**	**Thickness (mm)**	**LMA (g m** ^**−2**^ **)**	**δ** ^**13**^ **C (‰)**	**C:N**	**WD (g m** ^**−3**^ **)**
Mangata *S*. *acuminatum*	0.68 (0.01)	0.39 (0.01)	5.92 (0.55)	0.63 (0.04)	319.05 (17.40)	−27.38 (0.23)	25.76 (2.90)	0.62 (0.00)
Kurkara *A*. *decaisneana*	0.92 (0.00)	0.58 (0.00)	2.65 (0.13)	1.00 (0.02)	530.50 (10.85)	−25.76 (0.25)	37.44 (1.44)	0.66 (0.01)
Altarpa *E*. *gamophylla*	0.84 (0.00)	0.51 (0.00)	22.99 (0.76)	0.44 (0.01)	270.76 (5.92)	−26.29 (0.45)	37.00 (1.40)	0.62 (0.01)
Tjanpi *T*. *pungens*	0.90 (0.01)	0.52 (0.01)	1.41 (0.04)	0.85 (0.02)	352.97 (9.15)	−14.79 (0.12)	45.28 (2.69)	Non-woody
Wanari *A*. *aneura*	0.79 (0.01)	0.46 (0.01)	0.90 (0.03)	0.81 (0.01)	422.40 (7.87)	−26.77 (0.36)	24.15 (1.90)	0.72 (0.01)
Ilykuwara *A*. *kempeana*	0.73 (0.01)	0.48 (0.01)	4.96 (0.11)	0.55 (0.01)	307.33 (5.42)	−26.07 (0.44)	20.67 (0.60)	0.76 (0.01)
Pukara *A. maisonneuvei*	0.83 (0.01)	0.55 (0.00)	0.01 (0.00)	0.65 (0.01)	292.94 (4.12)	−27.00 (0.27)	54.75 (3.28)	0.81 (0.01)
Kaliny-kalinypa *G*. *eriostachya*	0.88 (0.01)	0.58 (0.01)	8.57 (0.31)	1.02 (0.01)	564.22 (13.09)	−24.73 (0.27)	45.63 (4.30)	0.70 (0.02)
Kampurarpa *S*. *centrale*	0.69 (0.02)	0.23 (0.01)	4.17 (0.28)	0.76 (0.01)	168.96 (8.99)	−28.89 (0.22)	16.39 (1.95)	Non-woody

See Table 3 for explanations of each trait.

### Data analysis

#### Broad trait patterns

All data analyses were conducted using RStudio (Posit team, 2025; R version 4.3.1). Bayesian multinomial logistic regression models were fitted using the brms package (Bürkner, 2017) in R to investigate which leaf traits best predict dieback severity in both seasons. Dieback was modelled as a categorical response variable with species-level data aggregated into three dieback levels: low, moderate, and high, with high dieback set as the reference category. Five leaf traits measured across both seasons—RWC, LDMC, LMA, leaf thickness and leaf area—were used as predictor variables. All traits were scaled and centred (mean = 0, divided by standard deviation) prior to analysis. To account for potential variation among habitat types, habitat was included as a group-level effect (random intercept).

Separate models were run for spring and summer to assess whether particular traits were more predictive of dieback severity under non-stressful (spring) versus stressful (summer) conditions. Models were fitted using four Markov Chain Monte Carlo (MCMC) chains with 4000 iterations each (including 2000 warm-up iterations). Convergence was evaluated using trace plots and the potential scale reduction factor (R^), with all models showing good convergence (R^ close to 1). Posterior predictive checks confirmed adequate model fit, with observed data within the spread of the simulated (posterior predictive) distributions. Trait effects were considered significant when the 95% confidence interval for the estimated log-odds did not include zero. Full model results are provided in Table S2.

#### Species-level trait patterns

To examine species-level shifts in leaf traits, we calculated trait differences (Δ trait) by subtracting summer from spring values. Species were also grouped by dieback severity class (three species per class) to explore broader trait shifts among groups. For species-level comparisons, linear models were used with trait as the response and species as the predictor. For dieback class comparisons, linear mixed-effects models were used with dieback class as the predictor and species as a random effect. Pairwise comparisons were then made for species and dieback class models using the *emmeans* package in R (Lenth, 2023), with Tukey HSD applied to estimate pairwise differences, with p-values adjusted for familywise error. Model residuals were assessed using the *ggResidpanel* package (Goode and Rey, 2019). Arcsine transformations were performed on RWC and LDMC as both are proportional data, whereas cube-root transformations were applied to other traits where log transformation is not appropriate due to negative values of Δ trait. Where assumptions of normality were not violated, or a transformation did not result in normally distributed residuals, we did not transform data to allow a more ecologically relevant and intuitive result to be interrogated. For traits only measured in spring (e.g. δ^13^C, C:N ratio, WD), a similar approach was applied using spring data only.

To assess the influence of species identity, habitat type, season and their three-way interaction on trait variation, we fit linear mixed-effects models using the lmer function from the *lme4* package in R (Bates *et al.,* 2003). We conducted analysis of deviance (Type III Wald chi-square tests) on each mixed-effects model. Trait values were log-transformed when residuals were not normally distributed to improve model fit. Random effects included individual ID (to account for intraspecific variation), plot ID (for spatial effects), plant health rating (qualitative health estimates) and plant size (relativized within-species size score). Random terms explaining negligible variance were removed as necessary based on singularity checks from model summaries.

## Results

### Extent of seasonal change varied among traits and dieback severity classes

To determine if broad dieback category classes differed in leaf traits and if those relationships shifted over seasons, we explored the seasonal change in traits with species grouped into dieback severity class. In spring when conditions were relatively more benign, leaves of low dieback species were typified by maintaining relatively high RWC (Fig. 2a, Table 5, 1.17–3.28 95% CI, Table S2), while being structurally dense (higher LMA, Fig. 2c, Table 5, 1.52–3.21 95% CI, Table S2) and having on average, thinner leaves (Fig. 2d, −2.24- − 0.78 95% CI, Table S2) than the other dieback categories (Table 5). For these spring samples, moderate and high dieback species also generally had similarly lower RWC (Fig. 2a, −0.61-0.32 95% CI, Table S2) and thicker leaves (Fig. 2d, −0.58-0.54 95% CI, Table S2), with low LMA best predicting high dieback over low and moderate dieback species (Fig. 2c, Table 5). In summer, after several months of hot, dry weather, the high RWC of low dieback species was maintained from spring, whereas moderate and high dieback species had considerably lower RWC (Fig. 2a, Table 5, 0.41–1.68 95% CI, Table S2). Unlike in spring, leaf thickness and LMA in summer were not significantly different between low and high dieback classes (Fig. 2c, −0.177-1.272 95% CI, Table S2), likely indicating that the differences in those traits within each dieback class are more species-specific and less generalizable. Overall, LDMC was not a good predictor of dieback class, with no significant trends in either season (Fig. 2b, Table 5, Table S2). The moderate dieback severity class had smaller leaf areas than both high and especially low dieback classes (Fig. 2e, −3.18-1.12 95% CI, Table S2, Table 5), with no large seasonal shift in leaf area observed.

**Table 5:** Model predictions of leaf trait levels for each dieback severity class across both spring and summer

	Order	
Trait	Spring	Summer
RWC	Low > Mod = High	**Low =** **Mod > High**
LDMC	Low = Mod = High	Low = Mod = High
LMA	Low = Mod > High	**Low <** Mod > High
Thickness	Low < Mod = High	**Low > Mod < High**
Area	Low > Mod < High	Low > Mod < High

Bayesian multinomial logistic regression was used to estimate the log-odds of all five traits predicting each dieback class. The relationship between trait value estimates between dieback classes is shown by the order in the season column. Significant (95% confidence interval of log-odds does not include zero) differences in predicted leaf trait levels between dieback classes are shown by > or < depending on the direction, with = representing a non-significant difference. A change in order of dieback classes between spring and summer is shown in bold. Separate models were created for each season, with habitat as a random effect. See Table S2 for the full statistical summary.

Next, to determine which species had the largest seasonal shifts traits within each dieback severity class, we quantified the direct seasonal change within an individual (Δ trait). Generally, species in moderate and high dieback severity classes exhibited greater seasonal reductions in RWC and increases in LDMC (Fig. 3a–d). However, due to large intra-specific variability in Δ RWC and Δ LDMC for most moderate and high dieback severity species, the reductions in these traits are not always statistically significant, warranting further analysis as to whether this individual-level variation reflects habitat variability. Kaliny-kalinypa (*G. eriostachya*) was the only high dieback species that did not have reductions in RWC in summer and was similar to all species in Δ RWC except the two other high dieback severity species—pukara (*Aluta maisonneuvei* subsp. *maisonneuvei*, *t* = 3.23, *P* = 0.04) and kampurarpa (*S. centrale*, *t* = 3.50, *P* = 0.02). For other traits, trends in Δ trait tended to be more species-specific with less generalizable trends between dieback severity classes. For example, all species had variable but generally reduced leaf thickness between spring and summer (Fig. 3g, h). However, low dieback severity species had non-significant reductions in LMA (Fig. 3e, f) compared to moderate (*t* = −2.19, *P* = 0.15) and high (*t* = −2.26, *P* = 0.14) classes, likely due to increases in, or maintenance of, leaf area between seasons, relative to moderate and high dieback species which tended to have reduced leaf area (Fig. 3i). Altarpa (*E. gamophylla*), a low dieback species with the largest leaf area, commensurately had the largest seasonal differences in leaf area. Across all species more generally, kurkara (*A. decaisneana*) exhibited small seasonal differences in all traits.

**Figure 2:**
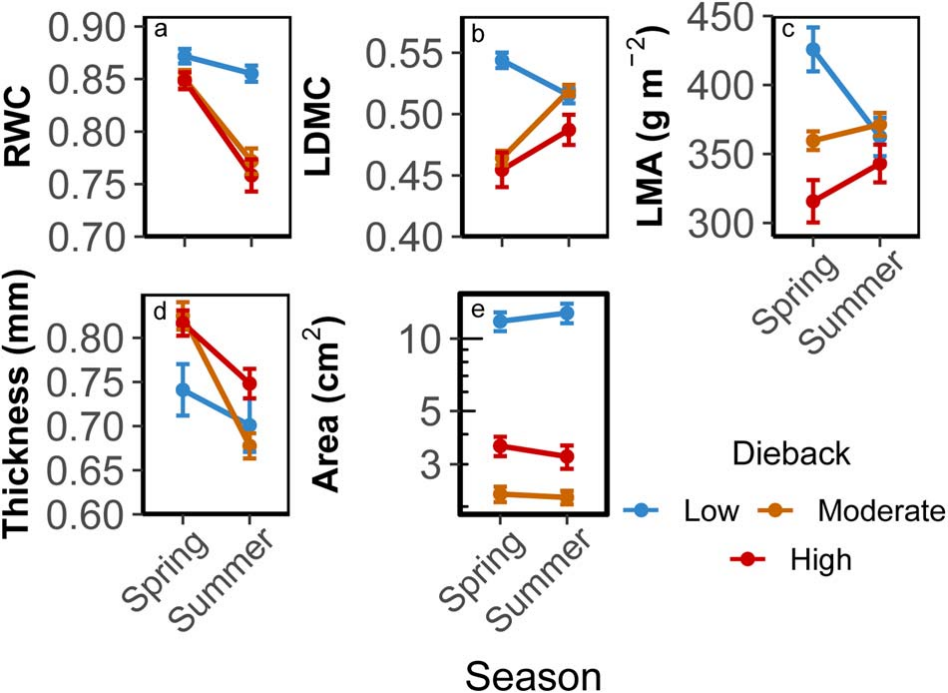
Seasonal differences in traits within each dieback severity class. Means (±standard error) are shown with lines connecting each dieback severity class’s seasonal mean. Bayesian multinomial regression was used to determine which traits best predict dieback severity class in each season (Table 5).

**Figure 3:**
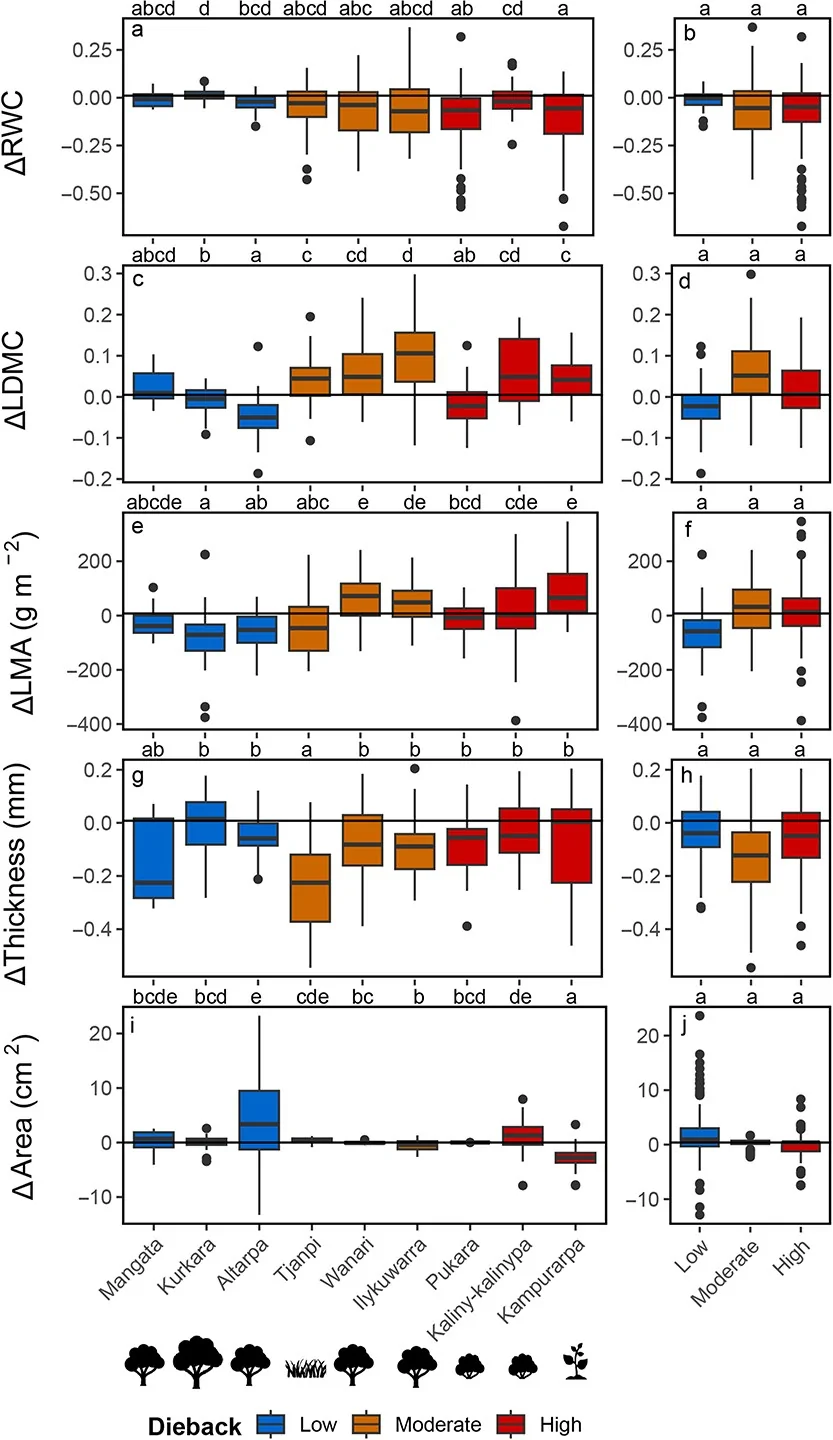
Seasonal differences in leaf traits among species and mortality classes. Summer measurements were subtracted from spring measurements for each individual. Positive values indicate that the trait value increased between spring and summer (and vice versa). Species data were aggregated across species by mortality class for the right-hand panels. Box and whisker plots show the median, 25th and 75th percentiles bound by the box, with the whiskers displaying the minimum and maximum values, plus outliers by points. Letters above each plot indicate significant differences, either between species (left column) or between dieback severity classes (right column) at *P* < 0.05 from Tukey HSD pairwise comparisons.

### Wood density is generally lowest in species with low dieback severity, but not water use efficiency or C:N ratio

Three of the traits, δ^13^C, C:N ratio and WD were measured only in spring because they provide more integrated measures of plant physiological and structural characteristics, rather than capturing seasonal changes in plant function as per the above traits (e.g. RWC). Although only seven of the nine species were woody, WD was lowest among species with low dieback severity (Fig. 4a). Both moderate dieback severity species had higher wood densities than all low dieback severity species with ilykuwara (*A. kempeana)* at 0.76 g cm^−3^ and wanari (*A. aneura*) at 0.72 g cm^−3^ (Fig. 4a). Pukara, with the highest mortality rate, had the highest WD at 0.81 g cm^−3^ (±0.01), although not significantly different from ilykuwara at 0.76 g cm^−3^ (±0.01, *t* = −2.80, *P* = 0.10, Fig. 4c). Kaliny-kalinypa, the species with the second highest dieback severity, had relatively low WD (0.70 g cm^−3^ ± 0.02) comparable to both low and moderate dieback species (e.g. kurkara, *t* = −2.09, *P* = 0.37, Fig. 4c).

**Figure 4:**
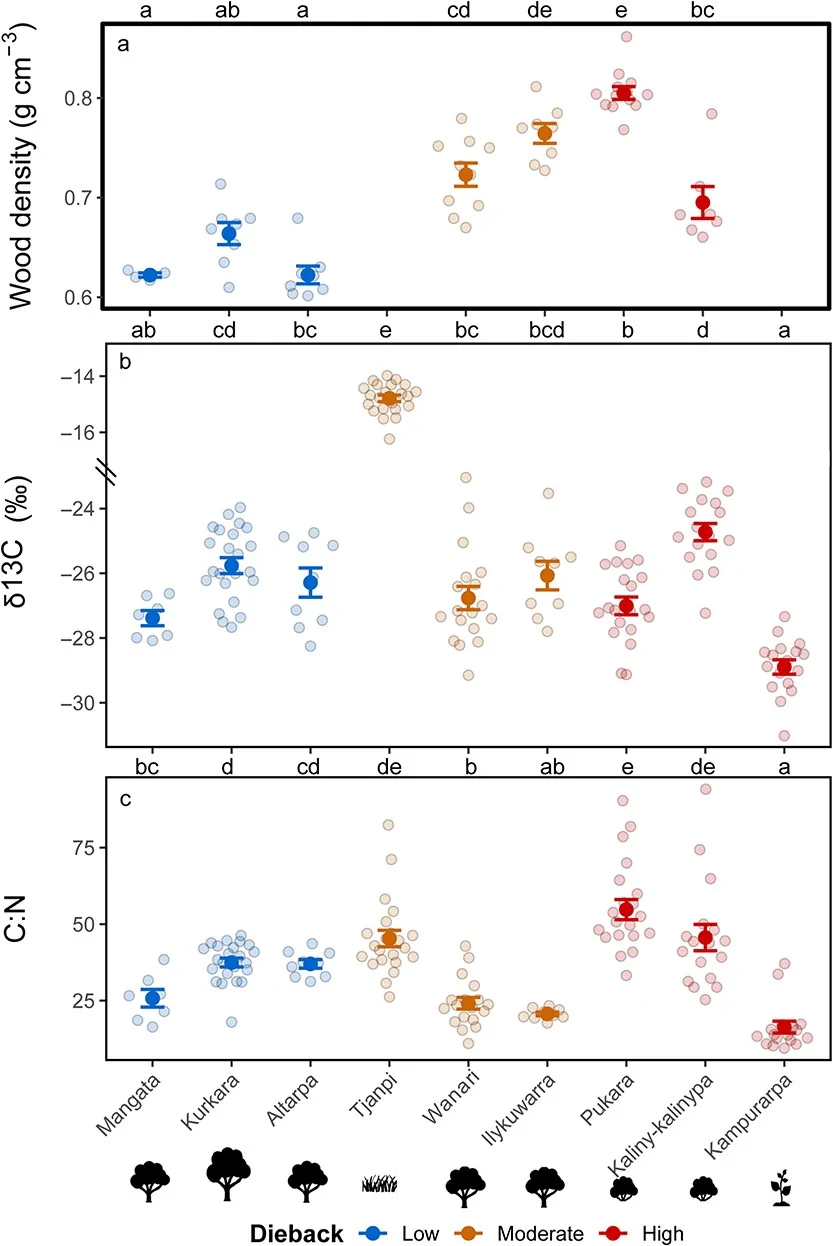
Wood density (**a**), leaf carbon stable isotope fractionation (**b**), and leaf carbon:nitrogen (C:N) ratio (**c**) values among species and mortality classes. Means ± standard error shown with background points representing each measurement. Letters above each plot indicate significant differences among species *P* < 0.05. Measurements are from spring only.

Of the C_3_ species, kaliny-kalinypa had the most conservative stomatal behaviour, with a δ^13^C value of −24.7‰ (±0.3), although not significantly different from kurkara (−25.8 ± 0.2‰, *t* = −2.82, *P* = 0.12) and ilykuwara (−26.1 ± 0.4‰, *t* = −2.97, *P* = 0.08, Fig. 4b). Kampurarpa, an ephemeral forb, had the least conservative stomatal behaviour (δ^13^C = −28.9‰ ± 0.2, Fig. 4b) and the lowest C:N ratio (16.1 ± 1.9, Fig. 4c), as expected for a fast-growing, non-woody species in the desert. The C_4_ spinifex species, tjanpi (*T. pungens*), had a δ^13^C ratio of −14.8‰ (±0.1, Fig. 4b), reflecting its photosynthetic pathway and water use efficiency. Variation in C:N ratio among species reflected life history in other ways, with the acacias (strong N-fixing species, ilykuwara and wanari) and the root hemiparasite, mangata (*S. acuminatum*), having lower C:N ratio values, indicating higher N concentrations in leaves (Fig. 4c).

### Within species, traits varied among habitat types as well as between seasons

Since Δ trait highlighted that some traits such as RWC and LDMC had high intraspecific variation (Fig. 3a and c), here we focus on how seasonal change in leaf traits differed among habitats, revealing significant species*season*habitat type interactions. Of these, RWC was particularly informative (Fig. 5). Low dieback severity species maintained high RWC between seasons (i.e. Fig. 2a) and commensurately showed no differences in seasonal change of RWC between habitats (Fig. 5a–c). Among these, mangata maintained RWC at a lower value of 67.6% across both seasons though we note our limited capacity to assess habitat differences for mangata due to a low sample size. Whilst overall reductions in RWC for tjanpi, a moderate dieback severity species, were low as expected for a C_4_ spinifex grass, those reductions were greatest in the pila (sand plain) habitat, at 13.4% (93.2–79.8%, *t* = −4.87, *P* < 0.01, Fig. 5d). Seasonal reductions in RWC for pukara, the species with the highest dieback, were the largest of any species, but only in pila, with an average reduction of 34% (88.3–54.7%, *t* = −11.1, *P* < 0.001), compared to 6% on tali (sand dune) and 3% on puti (woodland) (Fig. 5g). Although not statistically significant, kaliny-kalinypa, which also had high dieback, had comparatively low mean reductions in RWC of 3%, but slightly greater reductions in pila at 5.2% (Fig. 5h). Kampurarpa, which had low RWC at 69% on average, showed limited habitat-dependent seasonal differences in RWC, despite RWC dropping by 10% in summer (*t* = −4.24, *P* < 0.001, Fig. 5i).

**Figure 5:**
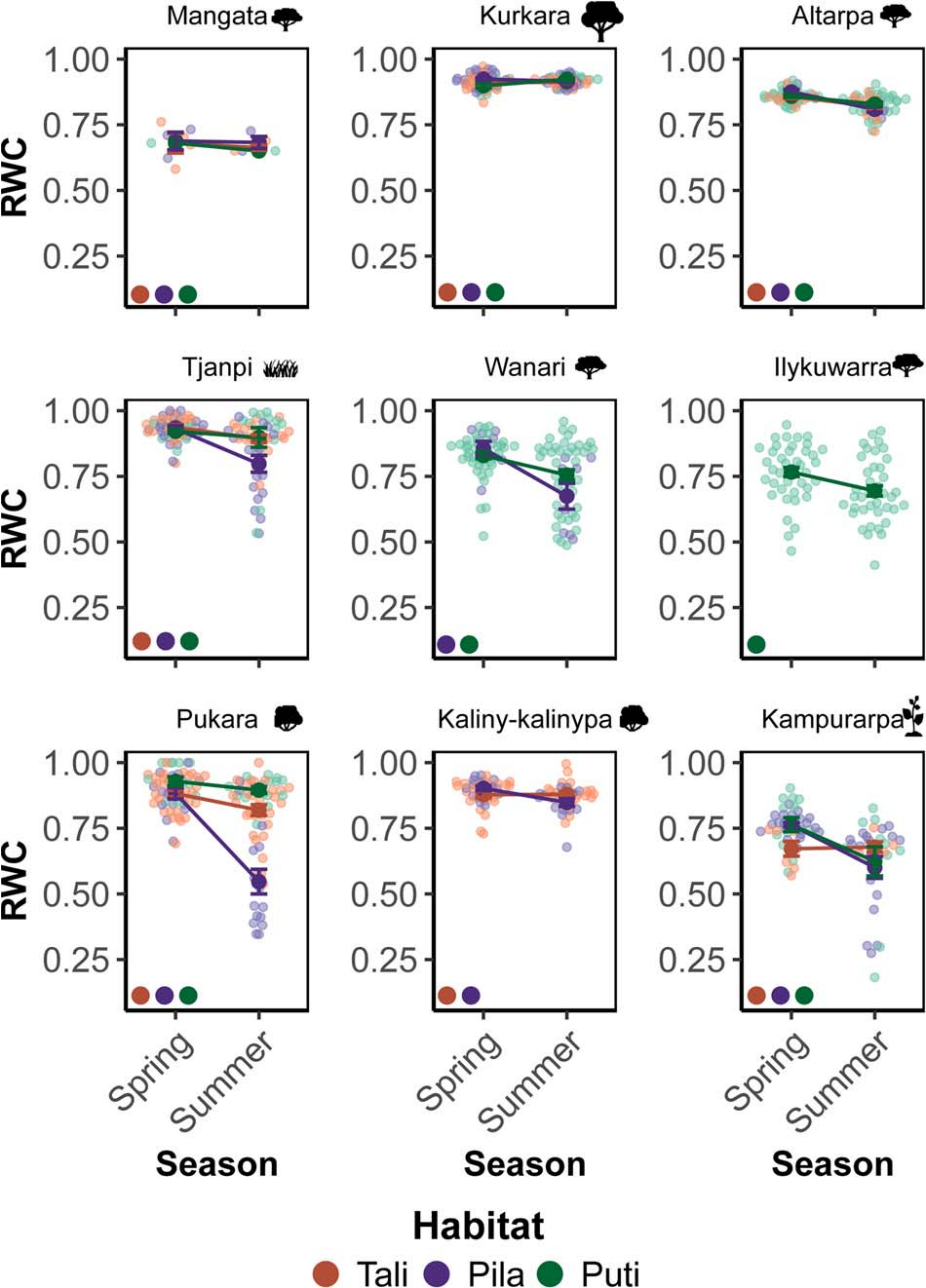
RWC (%) differences among habitats and between seasons for all species. Means (±standard error) are shown with lines connecting habitat means between each season. Species are organized by rows based on mortality class from low (**a**–**c**), moderate (**d**–**f**), to high (**g**–**i**). The circles in the bottom corner of each panel represent the habitats in which a species was measured: tali (sand dunes), pila (sand planes) and puti (shrubland, woodland).

Seasonal differences in LDMC were dependent on the species or the habitat, but there was no significant three-way interaction, rather there were significant two-way interactions with seasonal differences between species (i.e. species*season, χ^2^ = 144.6, *P* < 0.001, Table S3) having contributed somewhat more to LDMC variation compared to habitat differences between species (i.e. species*habitat, χ^2^ = 25.1, *P* = 0.005, Table S3, Fig. S2). Leaf thickness, LMA and area all had significant three-way interactions with species, habitat and season (Figs S3–S5). Similarly to LDMC, based on χ^2^ values, for LMA and area the species*season interaction contributed more to trait variation than the species*habitat interaction, indicating that temporal drivers of trait variation were more important than spatial drivers for these traits. However, the species*habitat and species*season interactions for leaf thickness had similar χ^2^ values, indicating that species-specific habitat differences are relatively more important than seasonal differences for species-level leaf thickness compared to the other traits.

## Discussion

We aimed to measure a suite of easily measured and accessible traits to characterize differences in physiological strategy and help explain variation in drought-induced mortality among ecologically and culturally significant Australian desert plant species. Generally, we found that species that had moderate and high mortality in a recent hotter drought event exhibited large reductions in RWC, or leaf hydration, between spring and summer, suggesting species using a drought-tolerating strategy were pushed beyond their physiological limits. All low dieback severity species exhibited a drought avoidance strategy, typified by maintenance of RWC in summer. Many species, especially in moderate and high dieback severity classes, exhibited seasonal shifts in traits that varied among the habitat types in which the individuals of the species were sampled. Overall, we observed a variety of physiological strategies, and have evidence for plant mortality caused by failure of both drought avoidance (possibly via carbon limitation) and drought tolerance (likely via hydraulic failure). Here we examine whether it is better to avoid or tolerate droughts in the Australian desert and consider the cultural and ecological significance of these differing drought strategies in the context of increasingly frequent hotter droughts.

### Is it better to avoid or tolerate hotter droughts in the Australian desert?

As has been demonstrated for other dryland plant communities, our data demonstrate that there are a range of alternative ecophysiological strategies among these co-occurring desert species (Laughlin *et al.,* 2023). In our study, the species with low dieback severity (mangata (*S. acuminatum*), kurkara (*A. decaisneana*) and altarpa (*E. gamophylla*)) all exhibited drought-avoiding strategies. Broadly, these species exhibited stable leaf RWC across seasons, suggesting conservative stomatal behaviour during a hot and dry summer (Jin *et al.,* 2023). This conservative stomatal behaviour can help maintain leaf water potentials above physiological thresholds, reducing the risk of hydraulic failure during extreme drought, although potentially at the cost of reduced carbon assimilation (McDowell *et al.,* 2022). To survive extreme hotter drought, we suggest that these low dieback, conservative water spending species may also rely on other traits we did not measure, e.g. deep roots, higher capacitance (or water storage) or increased carbon stores. Although these traits are recognized as important determinants of drought survival, they are difficult to measure and are consequently less commonly incorporated into trait-based predictions of mortality (Trugman *et al.,* 2021). The moderate and high dieback severity species, especially wanari (*A. aneura*), ilykuwara (*A. kempeana*) and pukara (*Aluta maisonneuvei* subsp. *maisonneuvei*), generally exhibited a drought tolerating strategy, typified by large reductions in RWC in summer and higher wood densities. Our interpretation is that these species are likely maintaining assimilation at more negative soil water potentials and may consequently accumulate greater proportions of native embolism and are, therefore, more easily pushed to hydraulic failure during hotter droughts, despite a presumed greater resistance to embolism (Hoffmann *et al.,* 2011).

Our inference in this study is based on a single seasonal comparison; thus it is important to consider how representative the observed differences within and among species are likely to be. We argue that our two sampling seasons provided contrasting conditions to compare seasonal responses across species and habitats. The winter conditions preceding our spring sampling (September 2023) were particularly mild with a large rainfall event in June (see Fig. S1a). The two months prior to summer sampling (March 2024) were mostly, but not entirely, rain-free with mean maximum daytime temperatures nearing 40°C, which represents average summer conditions. These temperatures are more than 5°C lower than the maximum temperatures recorded in the 2019 hotter drought, which precipitated the dieback event. We observed significant shifts in traits such as RWC across species and habitats in an average year, and we expect the observed plasticity is likely smaller than would be expected during an extreme year. Below, we explore in more detail the species-specific strategies within each dieback severity class.

#### Low dieback severity species: Mangata, kurkara, altarpa

Mangata is a root hemiparasite and therefore, so long as its host has access to soil water (Nge *et al.,* 2019), is able to maintain function even during extreme drought or heatwave conditions. The capacity to maintain water flow from its host is suggested via consistently low, but seasonally stable RWC values and low WD (Loveys *et al.,* 2001). In our study, the mangata were generally associated with another low dieback species, kurkara. Kurkara is thought to be an exceptionally deep-rooted species (Morton *et al.,* 2011); with adults that can access deeper soil water sources likely less influenced by surface-level soil moisture stress. In our study, kurkara exhibited relatively conservative stomatal behaviour, evident by the seasonally stable and high RWC and moderate mean δ^13^C value of −25.8‰. The water-use efficient cladode morphology of kurkara, where stomata in furrows are likely to decrease water loss, and drooping architecture that can increase convective cooling and reduce light interception (Curtis *et al.,* 2012), are both likely to aid in maintaining this species within its safe physiological thresholds. Here, we focused on adult kurkara; we note that what dieback did occur in this species was spatially variable (Godfree and Knerr, 2025) and observed to be biased towards younger individuals (Wright *et al.,* 2023), which may reflect the susceptibility of younger individuals to extremely low surface soil moisture. Further investigation into hydraulic and thermal vulnerability of kurkara, including quantifying key hydraulic traits such as the water potential at 50% loss of xylem conductivity (*P*_50_) and hydraulic safety margin, across its distinct life stages is currently underway and will allow a holistic understanding of what is driving dieback.

Altarpa exhibits conservative stomatal behaviour and maintains high RWC during summer. During prolonged and extreme drought, the large lignotuber carbon reserves of this mallee *Eucalyptus* species could offset reduced carbon assimilation and effectively avoid drought whilst maintaining function. Field observations showed that new leaf flush was present between spring and summer, resulting in large seasonal differences in leaf area and overall reductions in LMA among individuals. Therefore, timing of rainfall is likely important for the onset of new growth, which will determine leaf morphology in this species. Conversely, the lower LMA of spring leaves in this species could reflect a strategy to facilitate higher photosynthetic capacity (Han *et al.,* 2008) during short-term pulses of rainfall during the optimal growth period. With increasing interannual rainfall variability in central Australia with climate change, there will be greater and more frequent drought periods alongside more intense flash flooding rainfall (CSIRO, 2024). Therefore, if altarpa is able to withstand extreme drought through its conservative stomatal behaviour and large carbon reserves, having lower LMA may be an advantage for this species to take advantage of larger rainfall events.

#### Moderate dieback severity: Tjanpi, wanari, ilykuwara

Ilykuwara and wanari are good examples of drought-tolerating species. Typically, these species have high wood densities and high LMA and LDMC, all structural components that would increase ability to withstand greater water tensions during periods of stress (McDowell *et al.,* 2008). Additionally, RWC decreased in summer in both, implying that both species are sacrificing leaf hydration to sustain growth. Wanari has extremely high stem resistance to embolism (*P*_50_ = −11.3 MPa, Peters *et al.,* 2021). Clearly, the hotter drought in 2018–2019 in central Australia was extreme enough to push a large proportion of individuals in this drought tolerant species beyond its physiological thresholds. Although wanari was classed as a moderate dieback species on average at the landscape scale, dieback in this species was spatially variable (Wright *et al.,* 2023; Godfree and Knerr, 2025), consistent with spatial variation in factors such as stand density and topographic position that influence plant-available water during drought. While hydraulic traits may determine the physiological limits of wanari, variation in site conditions may influence the timing and severity of water stress experienced by individual plants (O’Brien *et al.,* 2017). Consistent with this spatial variation in dieback, we observed differences in physiology across habitat types, with wanari in pila (sand plain) showing greater reductions in leaf RWC in summer compared to individuals in puti (woodland). Although trait variation among habitats was relatively small, these differences may become more consequential under extreme hotter drought, when heat stress can exacerbate drought-induced damage and accelerate progression towards mortality (Marchin *et al.,* 2022). As wanari is known to be a strong competitor for shallow soil water (Nano and Clarke, 2010), a greater understanding of how geology, topography and stand structure influence soil water availability may better explain patterns of mortality across the landscape (Trugman *et al.,* 2021; Callahan *et al.,* 2022; Godfree and Knerr, 2025).

In contrast to wanari and ilykuwara, the third moderate dieback species, tjanpi (*T. pungens*), is a quintessential drought-avoider. It is a C_4_ hummock grass with high water use efficiency that maintains RWC in most habitats. Structural characteristics that likely enable this stable RWC include curled leaves and sunken stomata which together lead to lower stomatal conductance (Xian, 2021). At UKTNP, larger individuals in areas of greater tjanpi density exhibited significantly higher mortality (Wright *et al.,* 2023; Godfree and Knerr, 2025), which may indicate that intraspecific competition was a driver of mortality in this species. Indeed, in our study only the tjanpi in the pila (sand plains) where large individuals tend to occur in greater densities (Buckley, 1981) exhibited reductions in leaf RWC in summer.

#### High dieback severity species: Pukara, kaliny-kalinypa, kampurarpa

Pukara, a small myrtaceous shrub, was the species worst affected by the 2019 hotter drought, with a mortality rate over 90% (Wright *et al.,* 2023). In fire-prone Californian shrublands, species with the lowest vulnerability to embolism (i.e. highest WD, most negative *P_50_*) experienced the highest mortality during drought (Paddock *et al.,* 2013). This pattern is mirrored by pukara, which had both the highest dieback severity and highest WD. Paddock *et al.* (2013) suggested this apparent paradox may arise when more shallow-rooted plants cannot avoid severe dehydration during extremely low soil water availability, increasing drought exposure despite greater embolism resistance. Pukara also showed the largest reductions in leaf RWC during summer of all species, suggesting a reduced capacity to maintain leaf hydration under hot and dry conditions which may have accelerated the rate at which physiological thresholds were surpassed under the extreme hotter drought in 2019. Furthermore, pukara had spatially explicit patterns of dieback from the hotter drought, seemingly linked to topographic position along tali (sand dune) slopes (Wright *et al.,* 2023; Godfree and Knerr, 2025). For the individuals that survived, we measured large habitat-specific differences in maintenance of leaf RWC across seasons. To elucidate the mechanisms causing spatially explicit mass mortality in pukara, there is a need for a thorough investigation into topographic variability in microclimate, especially wind speed, vapour pressure deficit, and soil moisture, combined with experimental testing of the physiological impacts of heat and drought stress pukara hydraulic stress and thermal loads.

Kaliny-kalinypa (*G. eriostachya*), a shrub of similar size to pukara, also experienced widespread dieback across UKTNP. It is possible that a drought avoidance strategy in this species led to mortality from carbon limitation, rather than solely hydraulic failure associated with drought tolerance. Maintenance of high leaf hydration and δ^13^C values indicate conservative stomatal behaviour, and along with lower WD (i.e. higher stem vulnerability to embolism, Hacke *et al.,* 2001), these traits suggest this species ceases growth to avoid desiccation during mild stress (Choat *et al.,* 2018); characteristics more similar to all three low dieback severity species in our study. Furthermore, kaliny-kalinypa resprouts after fire, but Wright *et al.* (2023) found that individuals that had died during the 2019 hotter drought did not subsequently resprout within the 18-month period before sampling, potentially suggesting that belowground carbon reserves were depleted. However, the absence of resprouting does not demonstrate that carbon limitation directly caused mortality, and we cannot conclusively exclude hydraulic failure or other contributing factors, including biotic stress (McDowell *et al.,* 2022). Nevertheless, the combination of conservative water use, extreme hotter drought, and failure to resprout provides a plausible indication that carbon limitation may have contributed to mortality in kaliny-kalinypa. This species is therefore a candidate to test whether depletion of belowground carbon reserves contributed to mortality during hotter drought, a mechanism that has been suggested for resprouting shrubs experiencing drought stress during post-fire recovery (Pratt *et al.,* 2014).

Interestingly, the two small-statured shrub species with high mortality at UKTNP have seemingly opposing strategies to water use, with kaliny-kalinypa ceasing water loss at the cost to growth, and pukara sacrificing leaf hydration to maintain growth. Future measurements of non-structural carbohydrate reserves alongside hydraulic function could help distinguish carbon limitation from hydraulic failure as potential mortality pathways in these contrasting species (McDowell *et al.,* 2022). For both kaliny-kalinypa and pukara, we suggest burning areas of pila (sand plain) near tali (fire sensitive dunes) during cool winter times and establishing long-term monitoring plots, aimed at understanding the impact of abiotic conditions post-fire and post-germination on the fitness of individuals to hotter droughts (Pratt *et al.,* 2014).

Unlike all other species studied, kampurarpa (*S. centrale*) employs drought escape strategy. Being a desert herb, it has a facultative perennial habit, whereby, depending on conditions the year following germination, it can either persist and reproduce another season, or abort and rely on the next generation of germinants from seed (Van Buren *et al.,* 2021). Therefore, widespread mortality of kampurarpa is expected during extreme dry and hot periods. Perhaps of more importance to kampurarpa are the impacts of climate change on reproductive phenology and germination related to greater interannual rainfall variability (see a study on two similar species *Solanum oligacanthum* and *Solanum orbiculatum*, Milner *et al.,* 2023) and altered fire regimes (Ahmed *et al.,* 2006). Understanding the interactions between timing of rainfall, fire frequency and intensity, and increasing growing season temperatures, in combination with the ever-increasing abundance of the grassy weed buffel grass (*Cenchrus ciliaris*), which aids to further transform fire regimes in central Australia (Ryan-Colton *et al.,* 2024), will be an important topic of research at UKTNP and central Australia more broadly.

### Impacts of dieback on country and culture

Climate change is already having profound impacts on Anangu Country. The mass dieback event that followed the hotter drought in 2019 is an example of the scale of impact possible in a short timeframe, with uncertain consequences for vegetation communities as the climate continues to become hotter and rainfall becomes more variable. Drastic shifts in community composition and declines in biomass will continue to occur. For example, pukara naturally suppresses fire along dune systems, acting as a firebreak (Wright *et al.,* 2019), and field observations after the recent pukara mortality suggest that small tussock grasses are replacing pukara. Consequently, the dune field vegetation complex in central Australia may undergo significant restructuring, with replacement of fire-retardant species like pukara with fire-promoting species such as small tussock grasses and especially tjanpi (spinifex) along dune systems. Such shifts in vegetation could precipitate significant movement of dune systems in an otherwise stable system (Keith, 2017; Morton, 2022). Instability of dune system vegetation may also have impacts on rare and threatened fauna such as tjakura (great desert skink, *Liopholis kintorei*) and itjaritjari (marsupial mole, *Notoryctes typhlops*), which rely on the dune systems and pukara for habitat (Bennison *et al.,* 2014; Ridley and Schlesinger, 2023).

Areas of puti (acacia woodland) are important to Anangu for many reasons, including being an important habitat for tjala (honey ants) and maku (witchetty grubs). With mass death of wanari, the dominant species in puti habitat, we may see an encroachment of tjanpi (spinifex) and a retraction of woody puti species (Nano and Clarke, 2008). Wanari is an important species for Anangu used for ceremony, tools, and food, and supports high diversity of other important plants and animals (R. Okai, pers. comms.). Another potential ecological change to puti regions comes from the grassy weed buffel grass (*Cenchrus ciliaris*) which is a strong competitor and has been observed to persist and dominate understories beneath large kurkara (desert oaks). Anangu have expressed concern that buffel grass may decrease the competitive ability of important and large kurkara individuals (R. Okai, T. Guest personal communications).

Knowing more about the patterns of and mechanisms underpinning dieback at UKTNP is an important first step towards revising management approaches to protect Country. Anangu postulate that managing fire across the landscape via active cultural burning promotes ecosystem heterogeneity. We think these burning practices will likely help reduce the impacts of mass plant dieback, and even mitigate future dieback, particularly for tjanpi (spinifex) in the sand plains which is experiencing highest dieback severity in old, dense stands (Wright *et al.,* 2023), and promote regeneration of germination in pukara (Wright *et al.,* 2019). An ongoing research priority, therefore, is to conduct before-after, control-impact (BACI) experiments at UKTNP focussed on the potential for Anangu cultural fire as an active Country restoration tool. In addition, our results in combination with the spatial analyses underway (Godfree and Knerr, 2025) have application to explore whether there are any areas that, due to features such as topography, variation in groundwater depth, and microclimatic conditions, could serve as areas of drought refuge. Such areas, if they exist, would be a high priority for management. Finally, Anangu would like to increase their capacity at the local community nursery at Mutitjulu for growing culturally important species to maintain cultural connection, bank seed and re-plant important areas impacted by dieback. Our results increase our understanding of the functional ecology of these culturally significant species and will contribute to developing propagation and restoration protocols.

## Conclusions and key questions remaining

Large-scale plant mortality associated with climate change is already occurring at UKTNP and the rapid anthropogenic forcing of hotter droughts is likely to instigate subsequent events. The extent of seasonal decline in leaf RWC, a proxy for stomatal behaviour and water use strategy, was the trait that best predicted dieback severity across all species. All low dieback severity species mangata (*S. acuminatum*), kurkara (*A. decaisneana*) and altarpa (*E. gamophylla*) shared drought-avoiding strategies typified by stable RWC between spring and a hot and dry summer. These species also had distinctive characteristics that may help them achieve avoidance in different ways (hemiparisitism in mangata, deep roots accessing deep water sources in kurkara, and large carbon reserves in altarpa). In contrast, mortality in two high dieback severity species appeared to reflect contrasting consequences of water-use strategy: pukara (*Aluta maisonneuvei* subsp. *maisonneuvei*) had high WD and experienced large declines in leaf RWC, suggesting severe dehydration leading to death during the 2019 hotter drought, whereas kaliny-kalinypa (*G. eriostachya*) maintained relatively high leaf hydration with lower WD but may have experienced carbon limitation associated with prolonged conservative water use. In addition to conservative water use traits for kaliny-kalinypa, we point to the lack of resprouting observed by Wright *et al.* (2023) post-drought as further evidence to support carbon limitation as a potential pathway to mortality. The fact we found evidence for failure of both drought avoidance and drought tolerance strategies driving moderate and high dieback severity is consistent with the large unexplained variance in trait-based predictions of mortality (Anderegg *et al.,* 2016; Trugman *et al.,* 2021). In an arid Australian system, we have shown that a relatively short but intense hotter drought has the capacity to precipitate large-scale mortality in a range of species with either a tolerance or avoidance strategy. Our results provide further evidence of the need to consider species-specific strategies at temporal and spatial scales relevant to the local level, especially given mortality was variable spatially and across different vegetation types (as per Wright *et al.,* 2023; Godfree and Knerr, 2025).

Ultimately, while managing Country at UKTNP is mitigating detrimental impacts, the long-term impacts to Anangu, flora, and fauna are likely to be extensive without strong measures to halt and reverse climate change. The elevated and increasing contemporary temperatures and more frequent heatwaves combine to exacerbate the severity of natural droughts in arid Australia. A return of the prolonged droughts of the past (e.g. the Federation Drought 1895–1903, see Godfree, 2025) under future conditions of extreme heat could lead to transformational change to these culturally significant desert ecosystems. We hope that this study, and associated research in the region, provides a foundation for further investigation of resilience and vulnerability of plant species and communities to extreme events and supports potential identification of focal areas for conservation management. Moreover, we wish to continue to build shared ecological knowledge to empower Anangu, Uluru-Kata Tjuta National Park staff, and other researchers, to work together to manage these important landscapes in scientifically informed and culturally appropriate ways.

## Supplementary Material

Web_Material_coag079

## Data Availability

Due to cultural considerations and permitting, the data are currently not publicly available.
