# Supplementary material for: Physiological strategies explain mortality differences among ecologically and culturally significant Australian desert plants following a hotter drought
Source: Conserv Physiol. 2026 Oct 28;14(1):coag079. doi: 10.1093/conphys/coag079 (PMC13715015; doi:10.1093/conphys/coag079)
Supplement: Supplementary file 1 [file web_material_coag079.docx]

**Supplementary material**

Additional information for study sites.

For dune systems in central Australia, including those at UKTNP, Buckley (1982) found that soil moisture consistently increased from dune crest (top of tali) to swale (middle of puti). Represented as a percentage of water by weight in shallow soils (top 20 cm), on average crests had 0.6 % water content, mid-slopes 0.8 %, low-slopes 1.8 %, and swales 2.5 % (Buckley, 1982). Water content in deeper soil (1 m) followed similar trends, but was on average twice as high, especially in the low-slopes and in the swale. Fine soil fraction proportions also increase from crest to swale with very little silt and clay on dune crests, increasing to 3 % silt and clay on lower slopes and 5 % or more in the sandplain and swales (Buckley 1982). Nitrogen and carbon (mg kg-1) showed similar trends with both increasing between 3- to 10-fold from crests to swales (C from 500 to 2000 mg kg-1 and N from 50 to 200 mg kg-1, Buckley 1982). However, phosphorus showed no significant catenary pattern (20-30 mg kg-1 across sequence).

**Figure S1a**. Daily maximum temperature and rainfall total at Yulara Airport (NT) from January 2023 to April 2024. Maximum temperature is shown in red as a smoothed 5-day average. Daily rainfall total shown in blue. Sampling periods are shown in grey and were the first 3 weeks of September 2023 and the first 3 weeks of March 2024. Data accessed from <https://reg.bom.gov.au/climate/data/>. **Figure S1b**. Seasonal change in soil water content in the top 5cm (%) as measured by a time-domain reflectometry (TDR) meter at the base of individual study plants. Filled circles show the mean for each habitat (± standard error). The solid lines connect each habitat mean between seasons.

**Table S1.** Number of individuals sampled for each species, separated by habitat type.

| **Species** | **Pila** | **Puti** | **Tali** | **Total** |
| --- | --- | --- | --- | --- |
| Mangata  *Santalum acuminatum* | - | - | - | **8** |
| Kurkara  *Allocasuarina decaisneana* | 24 | 10 | 14 | **48** |
| Altarpa  *Eucalyptus gamophylla* | 5 | 25 | 15 | **45** |
| Tjanpi  *Triodia pungens* | 18 | 12 | 26 | **56** |
| Wanari  *Acacia aneura* | 8 | 44 | 0 | **52** |
| Ilykuwara  *Acacia kempeana* | 0 | 40 | 0 | **40** |
| Pukara  *Aluta maisonneuvei* subsp. *maisonneuvei* | 17 | 10 | 37 | **64** |
| Kaliny-kalinypa  *Grevillea eriostachya* | 15 | 0 | 24 | **39** |
| Kampurarpa  *Solanum centrale* | 22 | 14 | 7 | **43** |
| **Total** | **112** | **161** | **122** | **395** |

**Table S2.** Summary table of Bayesian multinomial logistic regression predicting mortality class from leaf traits. Separate models were run for each season of measurement to determine if certain traits are better at predicting mortality class during stressful (summer) and non-stressful (spring) conditions. For structure of models, see the methods section. Low, Mod[erate], High are mortality classes. Trait levels between mortality classes are determined to be significantly different if the 95% confidence interval does not intercept zero (emboldened). Random effect standard deviations reflect the variability in dieback probability attributable to habitat, with non-zero SDs (based on confidence interval not overlapping zero) suggest habitat influences the intercepts across dieback categories. All other estimates represent the log-odds of an individual plant being assigned to the Low or Moderate dieback class, relative to the High class. R̂ indicates model parameters converged successfully with all values < 1.01.

| **Parameter** | **Estimate** | **Error** | **95% CI** | **R̂** |
| --- | --- | --- | --- | --- |
| Spring (a) |  |  |  |  |
| *Random Effect (habitat)* |  |  |  |  |
| Low (intercept SD) | 1.971 | 1.200 | **0.654, 4.949** | 1 |
| Mod (intercept SD) | 2.106 | 1.184 | **0.798, 5.087** | 1 |
| *Fixed Effects* |  |  |  |  |
| Low (intercept) | -1.754 | 1.174 | -3.970, 0.731 | 1.001 |
| Mod (intercept) | -0.752 | 1.156 | -3.133, 1.564 | 1.001 |
| RWC (Low-High) | 1.931 | 0.555 | **0.903, 3.066** | 1.001 |
| RWC (Mod-High) | 0.126 | 0.25 | -0.379, 0.608 | 1.001 |
| LDMC (Low-High) | 0.751 | 0.487 | -0.179, 1.704 | 1 |
| LDMC (Mod-High) | -0.787 | 0.264 | **-1.309, -0.269** | 1 |
| LMA (Low-High) | 2.401 | 0.443 | **1.577, 3.284** | 1 |
| LMA (Mod-High) | 1.204 | 0.325 | **0.575, 1.839** | 1 |
| Thickness (Low-High) | -1.542 | 0.38 | **-2.313, -0.811** | 1 |
| Thickness (Mod-High) | -0.108 | 0.283 | -0.666, 0.45 | 1.001 |
| Leaf area (Low-High) | 1.533 | 0.324 | **0.943, 2.202** | 1 |
| Leaf area (Mod-High) | -2.215 | 0.533 | **-3.3, -1.224** | 1 |
| Summer (b) |  |  |  |  |
| *Random Effect (habitat)* |  |  |  |  |
| Low (intercept SD) | 1.561 | 1.055 | **0.425, 4.341** | 1 |
| Mod (intercept SD) | 1.930 | 1.076 | **0.705, 4.791** | 1 |
| *Fixed Effects* |  |  |  |  |
| Low (intercept) | -1.056 | 0.996 | -2.864, 1.171 | 1.002 |
| Mod (intercept) | -0.551 | 1.032 | -2.686, 1.562 | 1.001 |
| RWC (Low-High) | 2.555 | 0.491 | **1.65, 3.553** | 1 |
| RWC (Mod-High) | 0.457 | 0.152 | **0.164, 0.758** | 1.001 |
| LDMC (Low-High) | -0.293 | 0.382 | -1.023, 0.468 | 1.001 |
| LDMC (Mod-High) | 0.009 | 0.234 | -0.445, 0.461 | 1 |
| LMA (Low-High) | 0.553 | 0.369 | -0.177, 1.272 | 1 |
| LMA (Mod-High) | 1.067 | 0.315 | **0.457, 1.707** | 1 |
| Thickness (Low-High) | -0.171 | 0.339 | -0.828, 0.504 | 1 |
| Thickness (Mod-High) | -1.135 | 0.307 | **-1.743, -0.544** | 1 |
| Leaf area (Low-High) | 1.551 | 0.258 | **1.076, 2.079** | 1 |
| Leaf area (Mod-High) | -1.412 | 0.369 | **-2.159, -0.72** | 1 |

**Figure S2.** Leaf dry matter content (LDMC) differences among habitats and between seasons for all species. Means (± standard error) are shown with lines connecting habitat means between each season. Species are organised by rows based on dieback severity class from low (a-c) to high (g-i).

**Figure S3.** Leaf mass per area (LMA, g m^-2^) differences among habitats and between seasons for all species. Means (± standard error) are shown with lines connecting habitat means between each season. Species are organised by rows based on dieback severity class from low (a-c) to high (g-i).

**Figure S4.** Leaf thickness (mm) differences among habitats and between seasons for all species. Means (± standard error) are shown with lines connecting habitat means between each season. Species are organised by rows based on dieback severity class from low (a-c) to high (g-i).

**Figure S5.** Leaf area (cm^2^) differences among habitats and between seasons for all species. Means (± standard error) are shown with lines connecting habitat means between each season. Species are organised by rows based on dieback severity class from low (a-c) to high (g-i).

**Table S3.** Summary of analysis of deviance (e.g. ANOVA comparing goodness of fit, not sum of squares) applying Type III Wald chi-square tests on each mixed effects model below. Intercept is the reference level for all models (species = Altarpa, season = spring, habitat = pila), with significant intercepts for all models indicating that the reference group is not zero on average for all traits.

| **Trait** | **Effect** | **Chisq** | **Df** | **p** | **Variance** |
| --- | --- | --- | --- | --- | --- |
| **RWC** | Intercept | 242.6 | 1 | **< 0.001** |  |
| *Fixed* | Species | 213.8 | 7 | **< 0.001** |  |
|  | Habitat | 0 | 2 | 0.977 |  |
|  | Season | 2.3 | 1 | 0.126 |  |
|  | Species:Habitat | 64.5 | 10 | **< 0.001** |  |
|  | Species:Season | 71.4 | 7 | **< 0.001** |  |
|  | Habitat:Season | 1 | 2 | 0.621 |  |
|  | Species:Habitat:Season | 28.2 | 10 | **0.002** |  |
| *Random* | Plot |  |  |  | 12.28 |
|  | Health |  |  |  | 52.81 |
|  | Residual |  |  |  | 64.77 |
| **LDMC** | Intercept | 482.7 | 1 | **< 0.001** |  |
| *Fixed* | Species | 619.7 | 7 | **< 0.001** |  |
|  | Habitat | 0.2 | 2 | 0.901 |  |
|  | Season | 0.6 | 1 | 0.444 |  |
|  | Species:Habitat | 23.0 | 10 | **0.01** |  |
|  | Species:Season | 166.3 | 7 | **< 0.001** |  |
|  | Habitat:Season | 2.1 | 2 | 0.336 |  |
|  | Species:Habitat:Season | 13.0 | 10 | 0.223 |  |
| *Random* | Plot |  |  |  | < 0.001 |
|  | Health |  |  |  | 0.003 |
|  | Size |  |  |  | < 0.001 |
|  | Residual |  |  |  | 0.002 |
| **LMA** | Intercept | 2328.1 | 1 | **< 0.001** |  |
| *Fixed* | Species | 399.4 | 7 | **< 0.001** |  |
|  | Habitat | 2.9 | 2 | 0.238 |  |
|  | Season | 10.7 | 1 | **0.001** |  |
|  | Species:Habitat | 36.8 | 10 | **< 0.001** |  |
|  | Species:Season | 103.1 | 7 | **< 0.001** |  |
|  | Habitat:Season | 3.6 | 2 | 0.169 |  |
|  | Species:Habitat:Season | 22.7 | 10 | **0.012** |  |
| *Random* | Plot |  |  |  | 0.002 |
|  | Health |  |  |  | 0.021 |
|  | Individual ID |  |  |  | 0.005 |
|  | Size |  |  |  | 0.001 |
|  | Residual |  |  |  | 0.034 |
| **Thickness** | Intercept | 246.9 | 1 | **< 0.001** |  |
| *Fixed* | Species | 371.1 | 7 | **< 0.001** |  |
|  | Habitat | 5 | 2 | 0.082 |  |
|  | Season | 15.1 | 1 | **< 0.001** |  |
|  | Species:Habitat | 52.3 | 10 | **< 0.001** |  |
|  | Species:Season | 38.1 | 7 | **< 0.001** |  |
|  | Habitat:Season | 6.8 | 2 | **0.034** |  |
|  | Species:Habitat:Season | 43.1 | 10 | **< 0.001** |  |
| *Random* | Plot |  |  |  | < 0.001 |
|  | Individual ID |  |  |  | 0.005 |
|  | Residual |  |  |  | 0.014 |
| **Area** | Intercept | 198.1 | 1 | **< 0.001** |  |
| *Fixed* | Species | 4481.4 | 7 | **< 0.001** |  |
|  | Habitat | 5 | 2 | 0.084 |  |
|  | Season | 3.2 | 1 | 0.074 |  |
|  | Species:Habitat | 47.3 | 10 | **< 0.001** |  |
|  | Species:Season | 138.9 | 7 | **< 0.001** |  |
|  | Habitat:Season | 10.9 | 2 | **0.004** |  |
|  | Species:Habitat:Season | 44.5 | 10 | **< 0.001** |  |
| *Random* | Plot |  |  |  | 0.004 |
|  | Health |  |  |  | 0.131 |
|  | Individual ID |  |  |  | 0.025 |
|  | Size |  |  |  | 0.018 |
|  | Residual |  |  |  | 0.052 |
